# Three discipline collaborative radiation therapy (3DCRT) special debate: FLASH radiotherapy needs ongoing basic and animal research before implementing it to a large clinical scale

**DOI:** 10.1002/acm2.13547

**Published:** 2022-02-01

**Authors:** Patrizia Guerrieri, Naduparambil K. Jacob, Peter G. Maxim, Amit Sawant, Samantha J. Van Nest, Pranshu Mohindra, Michael M. Dominello, Jay W. Burmeister, Michael C. Joiner

**Affiliations:** ^1^ Department of Radiation Oncology Bon Secours Mercy Health Youngstown Ohio USA; ^2^ Department of Radiation Oncology Ohio State University Columbus Ohio USA; ^3^ Department of Radiation Oncology University of California Irvine California USA; ^4^ Department of Radiation Oncology University of Maryland Baltimore Maryland USA; ^5^ Maryland Proton Treatment Center Baltimore Maryland USA; ^6^ Department of Radiation Oncology Weill Cornell Medicine New York New York USA; ^7^ Department of Oncology Wayne State University Detroit Michigan USA; ^8^ Gershenson Radiation Oncology Center Barbara Ann Karmanos Cancer Institute Detroit Michigan USA

## 1 THREE DISCIPLINE COLLABORATIVE RADIATION THERAPY DEBATE SERIES

Radiation oncology is a highly multidisciplinary medical specialty, drawing significantly from three scientific disciplines—medicine, physics, and biology. As a result, discussion of controversies or changes in practice within radiation oncology must involve input from all three disciplines. For this reason, significant effort has been expended recently to foster collaborative multidisciplinary research in radiation oncology, with substantial demonstrated benefit.^1^, ^2^, ^3^, ^4^ In light of these results, we endeavor here to adopt this “team‐science” approach to the traditional debates featured in this journal. This article is part of the series of special JACMP debates entitled “Three Discipline Collaborative Radiation Therapy (3DCRT)” in which each debate team includes a radiation oncologist, a medical physicist, and a radiobiologist. We hope that this format will not only be engaging for the readership but will also foster further collaboration in the science and clinical practice of radiation oncology and developments thereof.

## 2 INTRODUCTION

FLASH radiation therapy (RT) delivers doses at ultra‐high dose rates (HDRs) generally understood to be >40 Gy/s, therefore a 2 Gy dose would be delivered in less than 50 ms. These ultra‐HDRs confer radioprotection by mechanisms yet to be clearly defined within the context of clinical radiotherapy. This radioprotection appears to dominate in normal tissues and less so (or maybe not at all) in solid cancers. This might allow an increase in the effective dose administered to the target malignancy. FLASH is not new. FLASH effects were originally reported in vivo over half a century ago as pointed out recently by Hendry.^5^ At the time however, the technology (and physics) available could not feasibly deliver such HDRs within the context of clinical radiotherapy nor provide a precise measurement of FLASH doses delivered to a patient. “FLASH” has been well named, evoking feelings of an almost explosive treatment of a cancer which won't be able to resist such an onslaught. So is FLASH destined for this exciting paradigm? Or eventually for the backwaters of radiotherapy? Or something in between? Right now we don't know because there are just not enough clinical evaluations or clinical trials to date. So the issue of a debate is not, and cannot yet be, FLASH “yes” or “no”, but rather, within the context of FLASH, do we proceed slowly into the clinic by first fully understanding the mechanisms (the how and why) by which FLASH appears to result in the effects seen so far both in the laboratory and in the limited clinical testing (For the motion)? Or, do we push forward more quickly with clinical implementation and trialing, with due caution but before knowing all the how and why of FLASH, accepting the possibility that some patients might not benefit from this approach (Against the motion)? So, hoping that you are well‐fired up now reading this, let us debate!

Arguing for the proposition will be Drs. Patrizia Guerrieri, Naduparambil Jacob, and Peter Maxim. Dr. Guerrieri is Board certified in Radiation Oncology in Italy and the USA, also an MS in Radiation Sciences and working in the field of radiation oncology since 1983 with current affiliation at the Department of Radiation Oncology, Bon Secours Mercy Health, Youngstown, Ohio. Her special expertise is in HDR brachytherapy, intensity‐modulated radiotherapy (IMRT), stereotactic body radiotherapy (SBRT), head and neck, breast and GYN cancers and she is author of publications, abstracts, and book chapters on GYN brachytherapy, altered fractionation, and brachytherapy in the elderly and contributor to the Radiation Oncology Encyclopedia and various editions of the Perez‐Brady book. Dr. Jacob is an Associate Professor in the Department of Radiation Oncology at Ohio State University Comprehensive Cancer Center, Columbus. His laboratory is particularly interested in radiation biodosimetry and developing strategies for protecting normal tissues from acute and delayed radiation toxicities. Dr. Maxim is a Professor of Radiation Oncology and Vice‐Chair of Medical Physics at the University of California, Irvine. His research focuses on the development of next‐generation radiation treatment technologies and studying their unique biological effects.

Arguing against the proposition will be Drs. Amit Sawant, Samantha Van Nest, and Pranshu Mohindra. Dr. Sawant is Vice Chair for Medical Physics in the Department of Radiation Oncology at the University of Maryland School of Medicine in Baltimore. He leads the physics components of the electron FLASH and proton FLASH research programs at Maryland. His other research interests include small‐animal image‐guided radiotherapy (IGRT), advanced motion management for thoracic and abdominal radiotherapy, image‐guided functional avoidance in radiotherapy, and modeling complex systems for radiotherapy treatment planning. Dr. Van Nest is a Postdoctoral Associate in the Department of Radiation Oncology at Weill Cornell Medicine in New York. Her research focuses on investigating mechanisms of radiation‐induced anti‐cancer immunity with particular focus on patient‐based platforms for optimizing immune activation and investigating the impact of radiation on the MHC‐I immunopeptidome. Dr. Mohindra is an Associate Professor of Radiation Oncology at the University of Maryland School of Medicine in Baltimore and Associate Medical Director of Radiation Oncology at the University of Maryland Medical Center. His primary area of clinical and research interest is in thoracic/lung, gynecological, and hemato‐lymphoid malignancies and evaluating treatment outcomes through institutional and population‐based databases, development of early phase clinical trials evaluating both radiation sensitizers and radiation toxicity mitigators, and evaluation of modern radiation techniques including proton beam therapy.

## 3 OPENING STATEMENTS

### 3.1 Patrizia Guerrieri, MD; Naduparambil Jacob, PhD; Peter Maxim, PhD (FOR)

FLASH‐RT looks like a dream coming true! It stands on the shoulders of standard delivery of radiation with the promise of widening the therapeutic ratio by relatively increasing the tumoricidal effects, while, at the same time, greatly reducing the side effect of this powerful tool called ionizing radiation. But we have witnessed and have heard the tales of a long story of innovation and failings. This story is more than a 100 years old and started with Madame Curie's discovery of the healing properties of radioactivity, and with the discovery of the tumoricidal effects of radioactive uranium. It took many trials to tame the beast, and it took pain and loss of lives. It took time, and, with time, the knowledge gained has allowed us to use it safely and to understand better its effects on live matter.

Up until about the 1990s, we studied and got used to the effects of what we now refer to as “standard fractionation” of a radiation treatment in what is equivalent to about 2 Gy/day. During the 1970s, we started studying a new radiobiological model, the linear‐quadratic (LQ) model that ultimately guided us to modulate the treatment with different fractionation schedules and be able to compare these to standard fractionation, in order to predict the therapeutic outcome and the related risk of side effects, according to dose, volume, and time. We have been using this model for the last 30 years; first, in the delivery of HDR brachytherapy and more recently to safely utilize hypo‐fractionation and SBRT in many different clinical set ups and primary tumors: from breast, to prostate, to lung, etc.^6^ We found this model successful in that, but we have also witnessed controversies and limitations especially in predicting the risk of ultra‐hypo‐fractionation or SBRT.^7^, ^8^ We have overcome the uncertainty of biological matter behavior in these extreme settings, by finessing the tools of technology, and by limiting the volumes of SRS, SBRT, etc., and by doing that, we have pushed the envelope of the tumoricidal action of XRT. It seems that we are now pushing this envelope even further with the invention, or re‐discovery according to some, of FLASH‐RT.^9^, ^10^ The question is once again, are we ready to do it safely? Are tools and models used thus far, to establish the potential of this technique, sound? And how rigorous are those analyses?

Although we took major strides in perfecting the delivery technique with the use of very sophisticated and expensive technology, how much do we really know of what is happening in the biological matter? We may have followed the established norms and rules and have used the available biochemical tools and multiple animal models for preclinical development.^11^, ^12^, ^13^ However, is the evidence obtained conclusive and strong enough to declare it safe to administer this type of ultra‐dose rate radiation to humans, for curing human cancers? We can see several problems with that.

First, we do not know exactly, at the molecular, cellular, or tissue level, how normal tissue sparing is achieved by FLASH‐RT. We are introducing a lot of physical complexity in inherently complex biological matter with a treatment that is based not simply on the volume of irradiation, but on pulses of high radiation on sub‐microvolumes.^14^, ^15^ We might be interfering with the biological matter in a way that is very different from the known mechanisms of action of photons or particles.

Second, we are basing our understanding of this technique on old radiation biology concepts like the GRID, a tool used from the 1960s on, for different practical purposes and that is currently being reevaluated in a more modern fashion. GRID and FLASH therapies have in common a highly heterogeneous dose distribution, very high prescription doses and an overall lack of experience among physicists and clinicians.^16^, ^17^

Third, the science behind FLASH effect(s) is largely unknown. Some may argue that promising benefits of FLASH have already been demonstrated in multiple preclinical models, and several hypotheses have already been developed. However, as of now, no single mechanism has proved to underlie, experimentally, the FLASH effect, unequivocally. It will likely take another whole generation of investigators to untangle all the biological mechanisms behind the FLASH effect. Indeed, impact of the levels of oxygen in radiosensitization is well established and reoxygenation is one of the classic four Rs that we learn in radiobiology. However, we do not know enough on the biokinetics and impact of varying levels of oxygen, in the context of FLASH‐RT, in a heterogeneous tissue. It is hypothesized that dose delivered at a rate higher than 40 Gy/s will result in normal tissue sparing, as oxygen depletion can be faster than reoxygenation in normal tissue. Has this hypothesis been positively tested in a controlled manner with quantifiable endpoints? We are relying on few animal trials, without a lot of follow‐up. The endpoints used for evaluating the effects in many animal studies such as fibrosis, cognitive and behavioral functions are qualitative or semi‐quantitative, at best.^18^, ^19^ We neither have a sound radiobiological model to guide us in the choice of fractionation, nor do we have, yet, a sound dosimetry system to fully understand all the dosimetric parameters associated not only to the atomic interaction of radiation with matter, but to other variables like instantaneous oxygenation and altered tissue response.^20^, ^21^

Fourth, concerning the physics of FLASH delivery, all the available data obtained thus far have been using small fields, that is pencil beams, etc. Is the FLASH effect still observable in large fields commonly used in actual clinical scenarios? What about the penumbra region, can we observe it there as well? How important is the penumbra and how “steep” must it be? What are the pulse structure, repetition rates, and other beam characteristics to obtain the optimal FLASH effect, and are these requirements both strictly necessary and sufficient? In terms of quality assurance, how do we monitor the dose accuracy, and how do we dose‐correct in case of beam‐interrupts or in‐transit beam instabilities?

The central question is how to translate this clinically; recognizing that in the past, our field had adopted new technologies before their biological mechanisms of action were fully understood. So how do we implement FLASH? Is it simply by developing new technologies (e.g., PHASER)? Is this something like a new and never‐seen drug with some kind of effect that is purely phenomenological? How much do we need to learn from preclinical studies concerning toxicity profile and anti‐tumor efficacies? Is the effect organ specific or universal for all tissue types and how different can it be *within* an organ? Is FLASH as effective in combination with systemically delivered agents?

So, are we risking imitating the try‐and‐fail pattern of the first decades of the 20th century, when many lives were lost to side effects and with solid and liquid second cancers caused by the unwise use of radioactivity? While we recognize the allure of a treatment that promises us to spare adverse events by more than 40%, we argue that we need more basic research studies, more randomized and blinded animal trials with good, longer follow‐up, showing differences with quantitative and measurable endpoints. As said, the clinical research has started already, and we have already the first patients irradiated by this technique. Is this the right way to proceed or do we need to take a step back and resist the pushes of the medical industry and its business agenda?

### 3.2 Amit Sawant, PhD; Samantha Van Nest, PhD; Pranshu Mohindra, MD (AGAINST)

Preclinical research plays a pivotal role in improving our understanding of the underlying disease and treatment response mechanisms, which in turn can inform the design and development of human clinical trials—the first step toward widespread clinical translation. Historically however, for a focal therapy such as RT, truly practice‐changing developments (e.g., SBRT, particle therapy, and image‐guided adaptive radiotherapy) have been driven in large part by technological advancements. Initial clinical experiences have then been followed by targeted in‐depth radiobiological testing to refine the clinical indication via improved mechanistic understanding. On the other hand, investigations that have originated primarily on the preclinical side, examples include tumor radiosensitizers and/or normal tissue radioprotectors, have still yet to see widespread clinical adoption. These simple observations form the basis of our argument against the proposition that FLASH‐RT *needs* ongoing basic and animal research before implementing it to a large clinical scale. In fact, the first clinical trial of proton FLASH in deep‐seated tumors (the FAST‐01 trial for symptomatic bone metastases) has already been activated and met its accrual target of 10 patients (NCT 04592887).^22^ We therefore postulate that given our understanding from preclinical investigations reported to date, safe clinical implementation of FLASH can be achieved in the form of large human clinical studies. The findings of these studies would then inform targeted preclinical radiobiological studies to test more focused hypotheses. This symbiotic approach can ultimately lead to much greater feasibility of and efficiency toward the clinical translation and widespread adoption of FLASH.

The current body of preclinical data, over several animal models and different tissue types (e.g., skin, lung, brain), has already consistently demonstrated that profound normal tissue radioprotection can be achieved via FLASH compared to that achievable in conventional dose rate RT, while maintaining iso‐effective anti‐tumor activity.^12^, ^18^, ^23^, ^24^, ^25^, ^26^ This collective evidence suggests that this FLASH effect is almost certainly reproducible in human patients, for which we have now at least one study with favorable outcome.^11^ Admittedly, an in‐depth mechanistic understanding of the observed differences between FLASH and conventional dose rates is lacking, with reports suggesting differential DNA damage/repair response,^27^ oxygen effect,^28^, ^29^ and proinflammatory signaling upregulation^27^ as possible explanations. Undoubtedly, further elucidation of these mechanisms will prove invaluable. However, taken together, the results to date strongly indicate that increased testing in larger clinical trials, with careful consideration on selecting patient cohorts and dose distributions, will more than likely be safe and will then enable us to ask more targeted preclinical questions.

After more than a century of treating patients using radiation, countless lives have been improved. In parallel, there have been continual improvements to our mechanistic understanding and optimal implementation of RT. The field of radiation oncology would not have evolved if, as a community, we had waited to completely understand radiobiology in preclinical settings before initiating large‐scale clinical treatments. In fact, successful clinical implementation can stimulate further radiobiological investigations. Arguably, the most prominent example is SBRT—more than a decade of clinical use of SBRT, establishing safety and efficacy, pre‐dated the extensive radiobiological research that has been conducted in recent years to refine utilization of SBRT. In a similar manner, clinical implementation of FLASH to the most obvious cohorts who could safely benefit would offer unique patient data. Human data are invaluable, indeed essential, since inter‐species variation in radiobiology is significant. It would also help dictate the most urgent and relevant preclinical directions, effectively focusing limited resources.

Signals of treatment effect in clinical studies also stimulate interest, and funding, for future research opportunities. For example, RT has been mainly used for local control of disease up until recently when evidence gained from clinical and preclinical experience identified the possibility of a systemic anti‐tumor response, motivating studies to further characterize the abscopal effect.^30^ While systemic response with RT could lead to a paradigm shift in the field, safe and effective use of RT which benefited many individuals was already achievable prior to this specific discovery. At the same time, deleterious effects of RT are also being uncovered, for example, evidence showing the potential for tumor cell migration following RT.^31^ However, this does not nullify the value of clinical RT to date.

An important point to consider is that even with improved mechanistic understanding at the preclinical level, for example, with murine models, the translation of FLASH to the clinic faces many barriers. There remains a big technological gap between the preclinical and clinical image‐guidance, treatment planning and treatment delivery technology. Thus, by delaying clinical implementation, we may be funneling extensive amounts of time and energy into preclinical characterization that may fall short the moment large‐scale clinical implementation is attempted. As an independent example, significant clinical motivation was gained for hyperthermia based on biological rationale. However, this treatment was sold‐short at the clinical point of implementation due to technical limitations. Similarly, in the case of cardiac radio‐ablation for the management of refractory arrhythmias, initial preclinical testing demonstrated efficacy of radiation in achieving ablation of the aberrant circuitry in heart. Clinical implementation was then limited by technological limitations in delivery of high‐dose RT to a moving target. Now that clinical evidence has demonstrated significant clinical benefit, a series of more focused preclinical radiobiological investigations has been triggered to further refine this as a treatment modality. We therefore believe that by similarly gathering extensive clinical evidence on FLASH‐RT, obtained within modern clinical radiotherapy settings, the preclinical approach can be guided to focus research efforts in a significantly more efficient and relevant manner. Furthermore, given that the current body of understanding suggests an encouraging advantage of FLASH‐RT in some patients, it would be a missed opportunity to wait for full characterization of the radiobiological underpinnings of FLASH and delay access to potentially life‐changing care. Therefore, tandem investigation of radiobiology led by extensive clinical experience and need is the ideal.

In summary, FLASH, even at its early stage of full radiobiological understanding, has more than enough justification to support large‐scale clinical implementation. At the same time, we should continue to investigate the underlying radiobiology that differentiates FLASH from conventional RT. The first reported clinical implementation of FLASH was made in 2019 to treat a multi‐resistant CD30+ T‐cell cutaneous lymphoma.^11^ This has been followed by the first clinical trial of proton FLASH in symptomatic bone metastases (NCT 04592887). While there is clearly a need to identify many more clinical settings where FLASH can yield positive outcomes, the long‐term strategy to successfully implement FLASH could follow the drug development pathway. The initial step which is common to both preclinical and clinical testing is developing the technical abilities to plan and treat animals and humans with FLASH. Having reached now the point of deliverable FLASH, clinical studies can test feasibility and safety in patients with superficial targets such as skin cancers which can be treated with electron FLASH or advanced/metastatic malignancies which need palliative radiotherapy to deep‐seated tumors using proton FLASH. This is akin to drug development through phase 1 dose‐finding studies in patients who have failed multiple lines of standard of care therapies. Clinical studies can then expand to efficacy studies to test responses (normal tissue and tumor) in the same population before moving on to formalized combinations with other treatment modalities, especially systemic therapy. Along the way of this escalation in clinical implementation, clinically driven radiobiological studies can be designed to continue to advance the therapeutic paradigm by defining the optimal dose rate, fractionation, and body site. FLASH‐RT has the potential to significantly improve therapeutic ratio while potentially reducing logistical and economical strain on a clinic. To conclude, continued progression of FLASH‐RT into clinical implementation via a staggered approach where clinical experience and technological limitations guide radiobiological research will allow optimal use of preclinical and clinical resources while allowing timely advancement of our ability to treat cancer.

## 4 REBUTTAL

### 4.1 Patrizia Guerrieri, MD; Naduparambil Jacob, PhD; Peter Maxim, PhD (FOR)

We acknowledge the argument of our colleagues about the need for integrating the advances in dose delivery technology in radiation oncology applications. However, we have not seen adequate preclinical radiobiological evidence, substantiating the readiness for transition of the FLASH technology to radiation oncology clinics. We need to keep the focus on the patients’ benefit first and ensure that the technology enhances therapeutic ratio through better sparing of normal tissues from acute and delayed toxicities, and with possibility for dose escalation to kill more cancer cells. It has been more than 7 years since Favaudon et al.^18^ published the qualitative or semi‐quantitative data from a mouse thoracic radiation model, suggesting reduced normal tissue toxicity such as fibrotic remodeling when doses are delivered at a very high rate. However, the validation of such sparing effects by FLASH on additional organs and organ systems in the follow‐up preclinical studies has been underwhelming. There is a need for demonstrating unequivocally the projected normal tissue sparing by FLASH on early as well as late responding organs, without significant protection in tumor tissues. A quantitative evaluation of toxicity endpoints in multiple organs showing a favorable risk–benefit ratio in at least two animal models is needed prior to making a “go” decision for using it in human patients.

One of the arguments made by our colleagues “against” the proposition is that, historically, methods for improved radiation delivery have been implemented in the clinic prior to, or even in parallel, with preclinical research. They seem to support a “symbiotic” approach where preclinical studies occur in parallel with clinical testing or use in humans, citing SBRT as an example, specifically particle therapy and IGRT. This is only true in part for IGRT and particle therapy, which are mere technological improvements based on established and known radiobiological pillars. Radiobiology of SBRT, for example, is based on the same biological principles of HDR brachytherapy, where large doses per fraction have been used since the 1970s. In our opinion, it is imperative that we first make sure that the approach is safe, beneficial, and at least does not worsen or incite additional adverse effects.

There are unanswered questions on the perceived benefit and therapeutic gain projected in FLASH‐RT. Indeed, a rational biophysical‐chemical model or theory around the principles of peroxyl radical reconstitution kinetics is used to explain the FLASH effect.^32^ Studies suggest that tissue sparing results primarily from local oxygen depletion due to the virtually instantaneous high flux of radiation into the tissue. However, crucially, it is not clear why such a mechanism must always protect oxygenated normal tissues, distinct from cancer tissues.^5^, ^33^ Thus there are great uncertainties on the extent of this protection, and hence overall therapeutic gain, with the varying physiological states of acute, chronic, and transient hypoxia, that are present in neoplastic tissues. The generalized notions such as the cancer tissues are hypoxic, still hold value; however, there are also large variations in oxygenation levels in normal as well as cancer tissues. Furthermore, the FLASH effect has been demonstrated only in three particular tissues: lung, skin, and brain, while its impact on other normal tissues is yet to be proven.^12^, ^18^, ^19^, ^34^

From a physics point of view, the best data available in the literature consider only the framework necessary to define dosimetric parameters like dose rate, pulse characteristics and frequencies, and total radiation time.^35^ Other dosimetry models and parameters developed seem to be based on matching data from experiments using electrons, which may not hold true to the level expected for particles such as protons and carbon ions.^20^

From a clinical point of view, a phase II trial was already started and accruing patients for FLASH‐RT with protons. Looking at the protocols however, it is difficult to comprehend how these will answer clinical questions or even help design follow‐up pivotal validation studies. The FAST‐Bone trial, in fact, should accrue just 10 patients with symptomatic bone metastases receiving a single dose of 8 Gy, mimicking an already effective and safe dose delivered with conventional modalities for pain palliation.^36^

Yet another argument of the opposing viewpoint is around the assumption that FLASH can potentially reduce the logistical and economical strain on a clinic. This argument, as well, is questionable or even controversial given the realities present in the current day world. To exemplify, let us take the case for an established prominent institution in a developed country such as United States where the capacity available for radiation treatments versus patient volumes is often skewed toward a high capacity with a relatively small volume of patients. Instead of focusing on reducing the machine usage time for an individual patient, it would be advantageous if we allocate resources for constructing facilities with additional sophisticated machines. Even in capitalistic societies like the United States, there is a need to maintain a viable healthcare ecosystem through proper resource allocation and retention of jobs. In the developing world or even in the underserved areas of the United States, FLASH‐RT would unlikely be a viable treatment option for most cancer patients in the near future.

### 4.2 Amit Sawant, PhD; Samantha Van Nest, PhD; Pranshu Mohindra, MD (AGAINST)

Both teams here agree upon the potential that FLASH‐RT represents. However, the two teams disagree about the timing of clinical translation. We believe that we are at a point where well‐planned clinical studies should commence, and the resulting clinical data should then be used to inform focused preclinical studies that answer progressively more nuanced mechanistic questions. Our opponents argue for a more traditional path to translation that requires an almost complete understanding of the mechanistic underpinnings of FLASH‐RT in the preclinical setting before embarking on human studies. We show in this rebuttal that our opponents’ arguments and proposed approach are informed by a selective and sometimes incorrect recollection of history, and will likely lead us along a path that is not only impractical but could also be counterproductive.

The opposing team first cites the early clinical use of radiotherapy following the discovery of radioactivity as a cautionary tale. There is no doubt that patient safety should be of paramount concern in all clinical radiotherapy, including FLASH. However, it is beyond naïve to compare practices from over a century ago with the complex process of introducing novel therapeutic modalities in the modern radiotherapy clinic—a process that involves clinical trials, multidisciplinary peer review, advanced multimodality imaging and image‐guidance, machine‐ and patient‐specific quality assurance and management, as well as longitudinal patient follow‐up. To quote the great American philosopher, Bob Dylan: “The times, they are a‐changin”!

Our opposing team's other major argument is based on inaccurate stating of historical facts. It is simply not true that the LQ model led to the clinical adoption of hypofractionation and SBRT. As noted in our opening statement, clinical testing of SBRT was independent of the LQ model and was enabled by improvements in technology that allowed the delivery of high‐dose fractions with high precision and reproducibility. In fact, the “classical” LQ model was not a mechanistic model of radiation response but rather an empirical fit to in vitro clonogenic survival of individualized cells,^37^ and failed to explain the excellent clinical outcomes of local control while limiting toxicity observed in high dose‐per‐fraction regimens.^38^ A similar story was associated with the evolution of HDR brachytherapy. In the early days of these high dose‐per‐fraction regimens, investigators predicted vastly increased OAR toxicity based on their then understanding of the LQ model.^39^, ^40^, ^41^ In fact, the positive clinical outcomes from these high‐potency regimens led to vigorous debate that subsequently enriched our mechanistic understanding of the radiation dose response.^41^

The remaining arguments presented by the opposing team essentially boil down to—we don't quite understand the radiobiology of FLASH and we have not adequately characterized the underlying physics. There is merit to this viewpoint, which has also been echoed in a recently published editorial by Buchsbaum et al.^36^ However, as we explain below, the approach proposed by our opponents will be highly inefficient and, in some instances, infeasible toward addressing these very issues.

First, we do not dispute that there are many unanswered questions about FLASH radiobiology. Preclinical models are essential for performing rigorous experiments in well‐characterized systems to expand our understanding of biological mechanisms. However, over the past several years, preclinical evidence on arguably the most clinically relevant endpoint for FLASH—normal tissue and functional sparing without significant loss of tumor control, has been steadily mounting across a wide range of preclinical animal models from zebrafish, to mice, to pigs, to companion animals (cats, dogs).^10^, ^42^, ^43^ Furthermore, despite impressive advances such as organoid models and patient‐derived xenografts, these preclinical systems, especially small‐animal preclinical systems (where the vast majority of preclinical FLASH research is being performed), can still only offer a limited recapitulation of a human patient. Specifically, in the context of radiobiological preclinical studies, radiation responses are highly dependent on the choice and source of cell lines, the choice of animal species and often the specific animal strain within the species, and the immune status of the animal model.^44^, ^45^ We believe therefore, that we are now at a point where additional exploratory preclinical research is going to be inefficient (in our opponents’ own estimate, it will take “a generation”!) compared to targeted preclinical studies that are more directly informed by clinical insights.

Second, from a clinical physics standpoint, the “parameter space” in which to optimize FLASH cannot be easily or accurately studied in small‐animal preclinical models. For widespread clinical translation of FLASH, we need to determine optimal dose *and* dose rate distribution within the irradiated volume, optimal fractionation, and optimal delivery techniques, beam arrangements and modulation. We also need to directly investigate many clinically critical aspects such as field‐size dependence of the FLASH effect for large and small fields, substructure dosing/sparing, complex tumor motion and interplay with surrounding organs, and point‐dose versus volumetric dose effects. None of these (except perhaps fractionation) can be recapitulated in small‐animal experimental designs.

Third, while we agree with our opponents on the need for FLASH‐specific measurement and quality assurance technology, and clinical practice guidelines, there is a rapidly growing body of work in this space. Independent groups have published on FLASH dosimetry (definition and measurement),^21^, ^46^ radiation safety,^47^ beam commissioning,^48^ and treatment planning.^20^ More recently, the American Association of Physicists in Medicine (AAPM) has instituted Task Group 359 for FLASH radiation dosimetry.

We acknowledge that there is much room to improve the mechanistic understanding of FLASH and its technology. However, a traditional, linear, preclinical‐to‐clinical approach is not only suboptimal but is very likely inadequate to achieve these goals. The last two decades have shown that successful clinical implementation in RT is not dependent on complete mechanistic understanding—if that were the case, we would not have made it this far in successfully treating hundreds of thousands of patients with RT. In our opinion, the current state of our knowledge and technology of FLASH is at a point where we can safely and intelligently move forward with carefully designed clinical studies. Doing so provides both opportunities for patients to access cutting‐edge and potentially life‐saving treatment while moving the field of FLASH forward in a timely way—refining future implementation and directing preclinical research in relevant directions.

We conclude with a reminder that the use of radiation for cancer treatment started within 10 years of the discovery of ionizing radiation. Imagine how many lives would have been lost had we waited for the new field of radiobiology to completely understand mechanistic effects of radiation and truly understand fractionation (which are topics for intense debate and research even now) before starting clinical treatments. Therefore, in contrast to our opponents, we believe that an iterative model, where clinical implementation of FLASH guides focused preclinical mechanistic research, which further feeds back into increased clinical utilization, is the only feasible path toward widespread implementation of this new, promising treatment modality.

## CONFLICTS OF INTEREST

Naduparambil K. Jacob is a consultant to Capture Collective Inc. The University of Maryland team (Amit Sawant and Pranshu Mohindra) participate in the Varian‐sponsored FlashForward Consortium.
